# Biological mechanisms of disease and death in Moscow: rationale and design of the survey on Stress Aging and Health in Russia (SAHR)

**DOI:** 10.1186/1471-2458-9-293

**Published:** 2009-08-13

**Authors:** Maria Shkolnikova, Svetlana Shalnova, Vladimir M Shkolnikov, Victoria Metelskaya, Alexander Deev, Evgueni Andreev, Dmitri Jdanov, James W Vaupel

**Affiliations:** 1 Federal Arrhythmia Centre, Moscow Institute of Pediatry and Surgery, Moscow, Russia; 2 Laboratory of Survival and Longevity, Max Planck Institute for Demographic Research, Rostock, Germany; 3 Department of Epidemiology of Non-Communicable Diseases, State Research Centre for Preventive Medicine, Moscow, Russia; 4 Laboratory of Demographic Data, Max Planck Institute for Demographic Research, Rostock, Germany; 5 Laboratory of Biochemistry, State Research Centre for Preventive Medicine, Moscow, Russia; 6 Laboratory of Biostatistics, State Research Centre for Preventive Medicine, Moscow, Russia

## Abstract

**Background:**

Prior research has revealed large differences in health and mortality across countries, socioeconomic groups, and individuals. Russia experiences one of the world's highest levels of all-cause and cardiovascular mortality, great mortality differences within the population, and a heavy burden of ill health. Psychological stress has been suggested as a likely explanation of health loss and premature death in Russia and Eastern Europe. However, physiological mechanisms connecting stress with health in Russia remain unclear since existing epidemiological data are scarce and limited to conventional risk factors.

**Method and Design:**

The survey on Stress Aging and Health in Russia (SAHR) is addressing this knowledge gap by collecting an unusually rich database that includes a wide range of reported information, physical and cognitive health outcomes, and biomarkers in a sample of Muscovite men and women aged 55 and older. The total planned sample size is 2,000 individuals. The sample was randomly selected from epidemiological cohorts formed in Moscow between the mid-1970s and the 1990s and from medical population registers. The baseline data collection was carried out from December 2006 to June 2009. Interviews and medical tests were administered at hospital or at home according to standardized protocol. Questionnaire information includes health, socio-demographic characteristics, economic well-being, cognitive functioning, and batteries on stress and depression. Biomarkers include anthropometry, grip strength, resting ECG, conventional cardiovascular factors of risk such as lipid profile and blood pressure, and other biochemical parameters such as those related to inflammation, glucose and insulin resistance, coagulation, fibrinolysis, and stress hormones. In addition to these measurements, SAHR includes dynamic biomarkers provided by 24-hour ECG (Holter) monitoring. This method continuously registers the beat-to-beat heart rate in naturalistic conditions without restrictions on normal daily activities. It provides information about heart functioning, including heart rate variability and ischemic and arrhythmic events.

Re-examination of the study subjects will be conducted in 2009–2011 and will focus on health, functional status, economic conditions, behaviors, and attitudes towards aging. The subjects are also followed up for mortality and non-fatal health events.

**Discussion:**

The SAHR will produce a valuable set of established and novel biomarkers combined with self-reported data for the international research community and will provide important insights into factors and biological mechanisms of mortality and health losses in Russia.

## Background

Between- and within-country disparities that had been decreasing during most of the twentieth century [1,2] began to increase in the 1980s [3-7]. In particular, the mortality gap between industrialized countries of Eastern and Western Europe has grossly increased [8]. In Eastern Europe, Russia is experiencing very high levels of all-cause and cardiovascular mortality and large differences in mortality between socioeconomic groups [5,9]. In addition to the toll of premature death, the Russian population has a high prevalence of ill health and low levels of healthy life expectancy [10,11].

Health behaviors are well established as factors of mortality in Russia [12], but along with conventional cardiovascular risk factors they can explain only a part of the excess death and socioeconomic mortality differences within the country [13-17].

It has been suggested that restricted life chances and psychosocial stress could be important contributors to poor health both in Russia and Eastern Europe [18-20]. Significant evidence connects relative deprivation and lack of life chances with exposure to various stressors [21-23]. Studies show the importance of effort-reward imbalance, hostility, social isolation, depression, low perceived control, uncertainty, and hopelessness [24-30]. For the Russian population, it has been suggested that the oppressive totalitarian regime as well as the drastic and poorly governed political and economic reforms of the 1990s were associated with high psychosocial stress and mortality increase [19,20,31]. It was found that mortality increases were greater in Russian regions that in the 1990s experienced larger socioeconomic shocks due to steeper pace of reforms, higher income inequality and unemployment [32,33].

In addition to classic questions on health behaviors and conventional cardiovascular risk factors, the SAHR program addresses the stress hypothesis through a range of questions on stress, coping, psychological and mental conditions.

How does stress, as reflected by these data, get "under the skin"? It is generally known that repetitive stress reactions connected with interruptions of the normal homeostatic process have the potential to suppress the body's adaptability. The concept of allostatic load was designed to reflect physiological toll caused by the cumulation of stresses [34,35]. It provides a possible basis for behavioral and physiological mechanisms by which genes, early life experiences, the living and working environment, interpersonal relationships, diet, exercise (physical activity), sleep, and other factors can affect the body's chemistry, neuro-physiological regulation and functioning over a lifetime. The allostatic load score is based on a set of biochemical parameters that express major multi-system functioning. Namely, these are lipid transport system parameters as markers of atherosclerosis; carbohydrate metabolism parameters as markers of metabolic syndrome; and markers of diabetes and insulin resistance, stress hormones epinephrine, norepinephrine, DHEA-S, and cortisol as markers of neuro-endocrine system disturbances, by measuring 12-hour hypothalamic-pituitary-adrenal axis activity and sympathetic nervous system activities. Coagulation system parameters are used as atherothrombosis risk markers, and inflammation parameters are used as indicators of atherosclerosis progression.

Point measurements of biomarkers, however, do not provide full information about real-life functioning that includes variations in bodily systems due to physiological cycles, diurnal rhythms, and responses to external influences. A review by Steptoe and Marmot suggests that dynamic measurement (or monitoring) that includes continuous recording of physiological response to exposures, has the potential to greatly enhance research opportunities [36]. Monitoring of subjects in ordinary settings, over hours or days, can provide especially valuable data that reflect not only characteristic levels of biomarkers but also patterns of their variation around these levels under normal life conditions. Studies involving blood pressure monitoring have produced a number of important results [37-40]. The SAHR program includes 24-hour heart rate (Holter) monitoring.

Elevated heart rate (HR) is an established risk factor of mortality and morbidity due to cardiovascular and other chronic diseases [41-49]. Although HR is simple to measure, its point measurements can be influenced by a variety of occasional environmental factors [50,51], limiting comparability and interpretation of results [52]. On the contrary, HR recorded over 24 hours has high reproducibility and is a better prognostic indicator than the traditionally measured resting heart rate in a hospital setting [48]. As a dynamic marker of challenge, HR is sensitive to external influences and responsive to stress.

Holter monitoring is a relatively inexpensive and non-invasive way of obtaining extensive data on the dynamics of cardiac functioning. The method is notable for the richness of data it provides. It produces large amounts of information by registering 70,000–100,000 consecutive cardio-cycles over a 24-hour period. These are multidimensional time series reflecting the organism's functioning via cardiovascular activity and its regulation by the sympathetic and parasympathetic influences of the autonomic nervous system. Holter tapes provide data on HR level, heart rate variability (HRV), and on cardiac events such as episodes of ischemia and various arrhythmias.

Reduced HRV is shown to be linked to negative emotions (anxiety and hostility), social isolation, and anger [53,54]. The research value of HRV analyses of Holter data collected from the general population has been recognized recently, and some promising results are already available. It has been shown that reduced HRV is associated with increased incidence of heart attacks in the general population aged 55 to 75 years with no initially registered pathology [55]. Another population study has demonstrated that elevated HRV indices are associated with higher risk of death among the elderly [56].

Circadian variations in heart rate reflect the sleep-waking pattern. Circadian changes predict life-threatening events such as myocardial infarction in the early morning hours [57-59]. Nocturnal heart rate elevations are recognized as a marker of severity of diabetes mellitus [52]. The ratio of average daytime to average nighttime cardiac rhythm is a stable characteristic of healthy individuals, and its deviations from a normal level have been found to be associated with disease progression [52,60-63]. Blunted circadian variation in the autonomic regulation of heart rate was found in US veterans with so-called Gulf War syndrome [64].

ECG events such as episodes of ischemia (ST depression) and arrhythmia (especially ventricular arrhythmia) are strong predictors of myocardial infarction, atrial fibrillation, heart arrest, and arrhythmic syncope [65].

### Aims of the Survey

The SAHR is designed to investigate physiological pathways of disease and death in a Moscow population that is exposed to high psychological stress and experiences high incidence of chronic disease and premature death. To address this general objective, the following specific aims are pursued:

- To conduct a biodemographic survey of men and women aged 55 and older, who reside in Moscow and closely resemble the socio-demographic structure of its general population, through collection of a wide set of major physiological function biomarkers, including Holter monitoring data.

- To perform descriptive analyses for estimating the prevalences and age patterns of health outcomes, diseases, health conditions as well as various biomarkers.

- To examine the links: (i) between health outcomes and biomarkers, with a focus on determining the incremental value of Holter monitoring as an additional source of research information; (ii) between biomarkers, socio-demographic characteristics, and stress; and (iii) sex- and age-related differences in stress-biomarker and health-biomarker associations.

- To conduct a re-examination survey of participants approximately 2.5 years after the baseline survey. To trace survey participants for mortality during another five years after the follow-up survey. To estimate feasibility of these data to predict morbidity and mortality.

- To study these longitudinal data, using statistical methods of survival analysis, with a focus on determining the incremental value of Holter information to predict morbidity and mortality.

- To prepare a user-friendly dataset that includes all the collected data and which can be used by the research community.

The general concept of the SAHR was worked out through a series of meetings of the International Study Team (IST) held at the Max Planck Institute of Demographic Research (MPIDR) in 2001–2006. The IST unites researchers from Germany, Denmark, Italy, Russia, and the United States. Sampling procedures, study questionnaire modules, procedures for realizing the biomarker collection, and other components of the study logistics were tried out in the Moscow Pilot Study (MPS) of 201 individuals aged 65 and older that was carried out in 2003–2004. The MPS outcomes indicated that the dynamic Holter parameters can be linked to perceived health, cognition, and stress and can potentially add value to the known risk factors [66]. In 2004–2005, these outcomes were used for planning the main survey.

The survey on Stress Aging and Health in Russia was launched in September 2006 as a core part of the project "Biodemography of Disease and Death in Moscow" [67] (Biodemography of Disease and Death, 2008). The project is a collaborative effort of Duke University (Durham, USA), the Max Planck Institute for Demographic Research (MPIDR, Rostock, Germany), and the State Research Centre for Preventive Medicine (SRCPM, Moscow, Russia).

### Stages of SAHR

The SAHR data collection program covers five project years. It began in September 2006 and will be completed by the end of June 2011. The initial recruitment and baseline survey have taken place, starting December 2006 and to be completed by the end of June 2009. The re-examination survey will be performed in 2009–2011.

Follow-up of the study participants for mortality along with telephone tracing of participants is being carried out over the 2008–2011 period. It is planned to continue with the mortality follow-up of SAHR participants until at least the end of 2016.

## Methods and Design

### The study sample

As do many studies, SAHR faced an inevitable tradeoff between the collection of sophisticated biomedical data for every individual and the sample size. The *sample size *was estimated using statistical patterns provided by previously available data: the MPS data and data on the survival of Moscow epidemiological cohorts (MONICA and Lipid Research Clinics cohorts specified in the next paragraph). We checked whether a sample size of 1,800 or more individuals aged 55 and older – similar to the statistical distributions and associations of the MPS sample – would be sufficient to estimate quantities and regression coefficients with a power of at least 0.90 with statistical significance p < 0.05. The findings confirmed that relative changes of about 5% between values of the principal health and stress variables measured at the baseline and at the re-examination surveys would be statistically significant. Monte-Carlo simulations confirmed the sample size for the coefficients of regressions connecting measures of health and psychological stress with risk scores based on conventional and Holter biomarkers. Using the survival data of the Moscow epidemiological cohorts, we also found that it would be possible to obtain statistically significant (p < 0.05) estimates of the coefficients of proportional hazard regressions connecting mortality to conventional biomarkers with statistical power exceeding 0.90. In the interest of descriptive analysis, we decided to include at least 100 males and 100 females in every five-year age group from 55–59 to 75–79 and at least 80 males and 80 females aged 80 and above. In addition, each of the age groups 55–64, 65–74, and 75 and older should constitute about one third of the sample.

The SAHR sample comprises *two unequal parts*: a random selection of members of past epidemiological cohorts listed in the registry of the international studies' participants of the SRCPM in Moscow and another random selection from the Moscow Outpatient Clinics registry. *The first part *of 1,860 individuals was randomly selected from seven epidemiological cohorts of the SRCPM that were first screened beginning in the mid-1970s. Data collected from these cohorts have been used in various international projects in the field of cardiovascular epidemiology [68-71]. The seven cohorts are the Lipid Research Clinics (LRC1) of 1975–77, the LRC2 of 1979–81, the Multinational Monitoring of Trends and Determinants in Cardiovascular Disease first cohort (MONICA1) of 1983–85, the MONICA2 cohort of 1988–89, the "Shigan" cohort of 1990–91, the MONICA3 cohort of 1992–94, and the MONICA4 cohort of 2000–01.

The data collection programs of the seven studies included a nearly identical part related to collection of conventional cardiovascular factors such as blood pressure, cholesterol and lipid levels, weight, height, electrocardiographic symptoms of ischemia; self-reported information on diet, smoking, chest pain, and angina; and basic socio-demographic characteristics. At first screenings, members of the seven cohorts were aged 40 to 59. Initial sizes of the cohorts varied from nearly 4,000 individuals in LRC1 and MONICA1 to about 800 individuals in MONICA4. The biggest cohorts were formed in the 1970s and the early 1980s. The initial total size of the seven cohorts was 14,402. According to the follow-up data, on 1 January 2007 the number of cohort survivors living in Moscow was 8,128. Of this total, 6,221 individuals were aged 55 to 85 and were eligible for further selection to the SAHR (Table 1). From this universe, about 930 men and 930 women were recruited to form the first part of the Survey sample.

**Table 1:** Subjects of the seven cohorts eligible for recruitment to SAHR

Age, years	Males	Females	Total
55–64	1,315	965	2,280
65–74	1,395	847	2,242
75–84	1,109	436	1,545
85+	115	39	154

Total	3,934	2,287	6,221

The majority of individuals from the seven epidemiological cohorts were resident in Moscow before the second half of the 1980s. During the soviet era, migration to Moscow was very limited because of special administrative restrictions. However, migration to Moscow from other parts of Russia and from abroad has intensified since the early 1990s. According to the last all-Russia census of 2002, the proportion of people who moved to Moscow after 1985 constituted 7% of the total population aged 55+ [72]. To ensure that this part of the Muscovite population is present in the Survey sample, 70 men and 70 women who moved to Moscow after 1985 are to be added to the sample. These 140 subjects constitute the *second part *of the Survey sample. Prospective candidates were randomly selected from the Moscow Outpatient Clinics' registry.

In the MPS of 2002–04, the response rate was 67%. For the main SAHR, we expected a 65% response rate. So far (after two years of fieldwork), the response rate is 64%.

### Recruitment

Recruitment to the study was launched after the vital status and addresses of potential participants had been updated. The recruitment scheme for the SAHR consisted of three subsequent steps: preliminary notice by letter, telephone contact(s), and checking into the survey. Starting in December 2006, the project manager of the SRCPM in Moscow contacted prospective participants by letter, enclosing basic information about the aims of the study, its contents, and potential benefits such as a high-quality medical examination that could provide early diagnosis of any serious condition.

Approximately one week later, the written invitation was followed by a direct telephone call in order to learn whether the subject would like to participate in the study. For each candidate subject, phone contacts can last no longer than one month with telephone calls performed at least twice a week on different days and at different times, with registration of results of each call. For subjects who did not want to participate, their reasons were noted, if given. If the subject was ready to participate, a date for a visit to the hospital or a date for testing and interviewing at home was agreed upon. Participants were asked to prepare a 12-hr urine sample in a clean container before the first clinic or home visit, to be collected at the time of the first visit. All subjects gave their informed consent before the examination.

Those who were ready to participate in the survey but were not examined for three months or who missed the visit three times were included in the cohort anyway but regarded as non-responders. If a potential participant was unavailable, the project manager attempted to find out why the subject was absent and note the reason.

At the beginning of the hospital or home visit, participants are registered into the survey and receive their unique ID-numbers. Only specially assigned medical personnel of the Moscow survey team have access to actual participants' names. All data components and documents are linked by means of ID-numbers.

On the first day of examination, SAHR participants are offered a snack. They are also offered free blood pressure devices to be used for the home blood pressure measurements. Within one week after the examination, participants receive information about health problems detected by the tests and related advice for further diagnosis and treatment.

### Baseline survey

#### Interviewing

The SAHR questionnaire includes well-established questions and batteries. Many of questions were piloted in the MPS in 2002–2003. The questionnaire modules are listed in Table 2.

**Table 2:** Modules of the baseline survey questionnaire

Module 1. First contact by telephone (or face-to-face) with a respondent or a proxy informant	Module 2. Information about the interview
Module 3. Socio-demographic characteristics	Module 4. Household composition
Module 5. Childhood and early life conditions	Module 6. Health behaviors
Module 7. Health and quality of life	Module 8. Functional status
Module 9. Major life events	Module 10. Wealth, economic conditions, and income-generating activities
Module 11. Supportive relations and social capital	Module 12. Stress and locus of control
Module 13. Cynical distrust	Module 14. Depression
Module 15. Coping with stress	

The *socio-demographic characteristics *module (3) has questions on marital status, children, education, and occupational status. The questions on *household composition *(module 4) were adopted from the Household Questionnaire used in the Russian Longitudinal Monitoring Survey (RLMS) [73]. *Childhood and early life conditions *(module 5) are addressed by questions on place of residence in childhood, ethnic origin, relative wealth of parental family compared to others, nutrition in childhood, and parental education [74]. Similar questions are asked in the Health, Alcohol and Psychosocial Factors in Eastern Europe (HAPIEE) study [75].

The *health behaviors *(module 6) includes questions on smoking and about frequency and amounts of consumed alcoholic beverages. Taking into account the important role of alcohol in Russia, the CAGE four-question battery [76] is also used. Finally, there is a set of more detailed questions adopted from the Izhevsk Family Study [77,78]. These questions are focused on heavy and binge drinking.

The *health and quality of life *(module 7) includes the 36-item Short Form Health Survey (SF-36) [79]. It has questions on physical ability, emotional problems, bodily pain, general health status, vitality, social functioning, and mental conditions. *Functional status *is assessed (in module 8) by questions on comparative health and on diseases from a slightly modified standard 35-item list. Cardiac symptoms are described by the classic Rose test batteries on angina and the pain of possible infarction [80]. Cognitive functioning is assessed by the Mini-Mental State Examination (MMSE) [81] in the form that was used in the Longitudinal Study of Aging Danish Twins (LSADT) [82].

The list of *major life events *(module 9) is based on the Holmes-Rahe Scale [83] and is slightly adapted to the Russian context. The list includes various events, especially adverse ones that can potentially cause stress [84,85].

*Economic conditions *(module 10) are assessed by questions on relative wealth compared to others, property, household assets, sources of income, kinds of expenses that the household can afford, and the share of income spent on food and non-food items. These questions were used in many surveys including the Russian Longitudinal Monitoring Survey (RLMS) and the Russian Generations and Gender Program [86]. The *social capital *questions (module 11) related to help and support provided to others and by others and questions on trust in others were adopted from a survey on social capital in Russia by Rose [87].

*Perceived stress *(module 12) is evaluated by Cohen's Stress Scale [88], an additional set of questions on psychological stress at work and at home, and questions on financial stress [89]. Perceived ability to control circumstances of one's own life is captured by the locus of control questions that were used in studies of psychosocial stress and health in Eastern Europe [90,75].

*Hostility and distrust *(module 13) are assessed by the 8-item Cynical Distrust Scale, which has been often used in epidemiological studies [91,92]. *Depression *symptoms (module 14) are determined by means of the depression section of the Cambridge Mental Disorders of the Elderly Examination (CAMDEX) instrument [93], which was also used in LSADT [82]. Finally, *coping with stress *(module 15) is assessed by the Brief Cope scale [94], which allows the identification of 14 coping strategies (such as coping by seeking emotional support by others, giving up, using alcohol as a stress-reliever, etc).

#### Biomedical tests

Table 3 summarizes biomarkers measured in SAHR baseline and re-examination surveys. They can be split into seven groups: anthropometry, physical measurements, ambulatory (home) blood pressure, two groups of laboratory tests (blood biochemistry and urine biochemistry), office ECG, and 24-hour Holter monitoring. Finally, participants' photographs are taken.

**Table 3:** SAHR biomarkers and their use in baseline and re-examination surveys

Biomarker	Baseline survey	Re-examination
**Anthropometry**		

Height	x	x
Weight	x	x
Waist circumference	x	x
Hip circumference	x	x

**Physical measurements**		

Office SBP	x	x
Office DBP	x	x
Resting HR during 30 seconds	x	x
Right grip strength	x	x
Left grip strength	x	x

**Ambulatory (home) blood pressure**		

Day 1: SBP (morning, evening)	x	
Day 1: DBP (morning, evening)	x	
Day 2: SBP (morning, evening)	x	
Day 2: DBP (morning, evening)	x	
Day 3: SBP (morning, evening)	x	
Day 3: DBP (morning, evening)	x	

**Routine blood tests**		

Haemoglobin	x	
Cell counts	x	
Color index	x	
ESR	x	

**Blood biochemistry**		

Total cholesterol	x	
LDL cholesterol	x	
Triglycerides	x	
HDL cholesterol	x	
Glucose	x	
Glycosylated haemoglobin	x	
C-reactive protein	x	
Gamma-glutamyl transferase (GGT)	x	
Insulin	x	
Dehydroepiandrosterone sulphate (DHEA-S)	x	
Fibrinogen	x	
Activity of fibrinilysis (ECLT)	x	
Interleikin-6 (IL-6)	x	

**Urine biochemistry**		

Creatinine	x	
Cortisol	x	
Norepinephrine	x	
Epinephrine	x	

**Office ECG**		

Minnesota-coded records, characteristics of ECG patterns, ischemia events (ST-segment), arrhythmia events, defects of conduction, ECG heart rate	x	

**Continuous 24-hour ECG (Holter) monitoring**	x	

**Facial photography**	x	

In most cases, the medical examinations are carried out by trained medical personnel at the hospital (SRCPM). If potential participants are unable or unwilling to come there (12%), the examinations are carried out in the home by personnel with the same qualifications.

*Anthropometry *includes body height measured with a wall-mounted stadiometer, body weight measured with calibrated scales, waist and hip circumferences measured with a calibrated tape in the standing position.

*Physical measurements *include resting blood pressure (BP) measured according to standardized procedure [95] on right arm using a manual sphygmomanometer after a 10-minute rest. The first and the fifth Korotkoff phases are used to define systolic and diastolic blood pressures, respectively. BP is measured three times, and the averages are taken as the office SBP and DBP values.

*Grip strength *is a measure of isometric muscle strength that correlates with the strength of many other muscle groups. It is measured by a hand-held Smedley Dynamometer "Scandidact" (Denmark). The upper arm is held against the trunk and the elbow in 90 degrees flexion [96]. Three trials with brief pauses are performed for each hand, and subjects are encouraged to exert the maximal effort. The same grip strength instrument and similar protocols have been used in other studies such as the English Longitudinal Study of Aging (ELSA) [97], the two Danish twin studies, MADT and LSADT [82], and the Eastern European HAPIEE [75].

*Ambulatory or home blood pressure *protocol consists of repetitive BP measurements at home over three consecutive days. Omron automatic monitor HEM-712 is used. The self-measurements are made on the right arm, twice a day in the sitting position, in the morning and in the evening. The home BP results are not biased by the so-called "white coat" effect characteristic of the office BP. Some research suggests that they are also more reproducible, better predict future BP [98], cardiovascular events [99], and mortality [100].

*Blood tests *are taken on venous blood with amount about 12 ml, drawn from cubital vein after overnight fasting and sampled for the routine blood tests and measurement of fasting plasma concentrations of glucose, insulin, serum total and high-density lipoprotein (HDL) cholesterol, triglycerides, fibrinogen, glycosylated hemoglobin (HbA1c), dehydroepiandrosterone sulphate (DHEA-S), interleukin-6 (IL-6), C-reactive protein (CRP), and gamma-glutamyl transferase (GGT). Whole blood is used for routine blood tests to count white blood cells and to assess erythrocyte sedimentation rate (ESR) and HbA1c, which is measured by High Performance Liquid Chromatography Technique (ECLT). Glucose level is measured by the glucose oxidase method. Serum total cholesterol, HDL cholesterol, and triglycerides are measured enzymatically on an auto-analyzer "Airone 200" (Italy) using "Human" reagent kits (Germany). Serum LDL cholesterol is calculated with the equation of Friedewald [101].

GGT activity is measured by colorimetric kinetic method on the auto-analyzer "Airone 200" with the "Human" reagent kits. Fibrinogen is measured according to the Clauss method. Activity of fibrinolysis is estimated as a time of spontaneous lysis of euglobulin fraction clot. Serum levels of DHEA-S and of insulin are determined by radioimmunoassay. IL-6 level is determined by quantitative sandwich ELISA-technique. CRP concentration is measured by means of high-sensitive particle-enhanced rate immunonephelometry.

#### Urine biochemistry

The 12-hour overnight excretion of cortisol, epinephrine, and norepinephrine reflects basal non-stimulated level of activity of the hypothalamic-pituitary-adrenal axis and sympathetic nervous system. Free epinephrine and norepinephrine are measured by high performance liquid chromatography, while cortisol is measured by radioimmunoassay. Creatinine level is measured by photometric colorimetric test. Values of cortisol, epinephrine, and norepinephrine are expressed in micrograms per gram of creatinine excreted during the same overnight period. In this way, the values are adjusted for variation in absolute amounts of the urine that are related to variations in body size and other factors [102,103].

#### Resting ECG

The baseline standard supine 12-lead electrocardiogram with four consecutive cardio-cycles (25 mm/s) is performed, coded according to the Minnesota Code [104] and examined by two independent experts. Over the past two decades, the Minnesota Code, which provides a classification of electrocardiographic morphology, has become a universal standard for ECG classification in epidemiological studies [105].

#### Continuous 24-hour ECG monitoring

Holter monitoring is performed with the Schiller Holter System, manufactured by Schiller AG (Switzerland) in accordance with the guidelines of the ACC/AHA [106]. Holter recorders (3-channel MICROVIT MT-101 digital devices) and electrodes are fitted to the participant's body by a qualified nurse and are carried by participants over the next 24 hours. Quality of the ECG initial signal is checked by visual inspection through a special window on the recording device. ECG recording is not disturbing and causes hardly any changes in participants' normal daily activities. After copying the recorded ECG series to the MT Holter System, two types of data files are produced: binary file of continuous ECG time series and ASCII file of RR-interval time series. The participants also keep diaries briefly tracing their main activities as well as episodes of bodily discomfort, pain, other symptoms, physical efforts, beginning and end of sleep.

**Figure 1:**
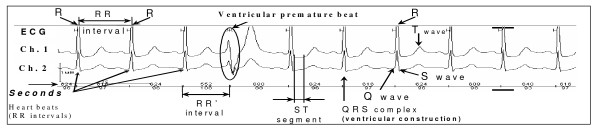
**A fragment of Holter series showing several ECG parameters and an ECG event (premature ventricular beat)**.

Preliminary cleaning of the Holter series is performed by trained cardiologists using the standard analytical toolkit of the Schiller System MT200 and MT210. Each Holter series is analyzed in semi-automated mode that allows the cardiologist to remove artifacts and to identify event patterns including arrhythmia, rhythm pauses, sinus tachycardia/bradycardia, and ischemia. Holter records with numerous artifacts and fewer than 18 hours of analyzable data are excluded from further analysis. Figure 1 demonstrates a fragment of Holter series, one ECG event (premature ventricular beat), and some ECG parameters.

In general, three types of biomarkers are estimated from the Holter ECG files. The first type reflects the heart rate, including its average, upper and lower extremes over 24 hours, and its day-to-night (circadian) variation. (The daytime period is defined as time between 8 am and 8 pm. The nighttime period lasts from 0 am to 4 am). Process of heart repolarization is described by QT and corrected QT interval values corresponding to the average, maximum, and minimum heart rates.

The second type of Holter biomarkers reflects the heart rate variability related to cardiac regulation by the autonomic nervous system. HRV parameters are calculated from the RR series. Only normal-to-normal (NN) intervals (intervals between adjacent normal heart beats) are included in the HRV analysis. A 5-minute interval of the RR series is included in the HRV analysis only if at least 80% of its RR intervals correspond to normal heart beats. Generally, HRV parameters are classified as either time or frequency domain parameters [107,108]. Methods for estimation of most of the time domain parameters are internationally standardized. The study uses SDANN (standard deviation of the averages of normal-to-normal intervals within all 5-minute segments); SDNN (standard deviation of all normal-to-normal intervals); SDNN index (mean of the standard deviations of all normal-to-normal intervals within all 5-minute segments); pNN50 (percentage of adjacent normal-to-normal intervals that vary by more than 50 ms); and rMSSD (the square root of the mean of the sum of successive differences of normal-to-normal intervals).

The frequency domain parameters of HRV are based on spectral analysis of RR series. Methods for their calculation are standardized to a lesser extent than those for the time domain parameters. The following frequency domain parameters are used: POWER – total power of the spectrum of tachogram in the frequency range ≤ 0.4 Hz; VLF – power in the very low frequency range ≤ 0.04 Hz; LF – power in the low frequency range 0.04–0.15 Hz; HF – power in the high frequency range 0.15–0.4 Hz; and LF/HF – the ratio LF/HF. While the HF component is considered as a pure index of efferent vagal activity, the LF component is influenced by the sympathetic control [107].

The third type of data is related to cardiac events. Arrhythmia events include supraventricular and ventricular premature beats, pauses of heart rhythm, periods of asystole, episodes of supraventricular and ventricular tachycardia, and other rhythm disturbances. Ischemia is detected on ST-segment trend built by the Holter System from the ECG series. This trend allows one to identify ST-segment deviations. The cardiac events are described by frequencies of their occurrences and durations of the abnormal episodes.

#### Facial photography

Photographs of participants' faces are taken from distance of 120 cm. They can be used for age assessment by assessors [109]. The assessed age is considered as a possible biomarker of successful aging.

### Implementation of the baseline survey

Before beginning the fieldwork, the study plans were approved by the Ethical Committee of the SRCPM in Moscow and the Institutional Review Board at Duke University in Durham, USA. Study planning, data processing and checks are performed jointly by researchers from MPIDR, SRCPM, and Duke University with advice from the study consultants.

SAHR questionnaires were translated from English into Russian and rechecked on the basis of the back translation. The Russian version was then tested in Moscow on a small number of respondents. Training sessions for the Moscow team members were completed in June-August 2006. Interviewing is performed by five trained interviewers with two additional interviewers doing interviews from time to time.

Fieldwork is performed by the Moscow SAHR team based at SRCPM. The team includes (for most of the time) a fieldwork supervisor, eight physicians, seven nurses, a statistician, and an epidemiologist. All team members have experience of prior epidemiological studies. Analysis of Holter series is performed by three qualified cardiologists.

The survey procedure includes a fixed sequence of steps and lasts two days. Most of the tests are performed on the first day. For cortisol and creatinine measurements, participants are asked to collect the first portion of overnight urine (excreted between 8 pm and 8 am) over the night prior to the first-day appointment. A second portion of urine for catecholamines is excreted between 8 pm and 8 am overnight following the first day of examination. The second urine portion and the Holter recorder are returned to the SAHR team on the second day. The completed home blood pressure table is sent to SRCPM within four to five days by postal mail.

The first-day steps are:

- Signing of informed consent

- Registration in survey and issue of personal IDN

- Face-to-face interview

- Collection of first 12-hour urine portion for creatinine and cortisol measurements

- Venous blood drawing

- Eating a snack

- Office measurements of blood pressure and heart rate

- Grip strength measurement

- Instruction on how to measure home blood pressure and provision of (free) blood pressure gauge

- Office ECG and anthropometric measurements

- Installing of Holter recorder on participant's body

- Taking participant's photograph

- Providing container for second urine portion and envelope with home blood pressure table

The second-day steps are:

- Returning second urine portion to SRCPM

- Removal and return of Holter recorder to SRCPM

### Data processing

For each study participant, the whole contact process (dispatch of the invitation letter, telephone calls, and results of negotiations) is coded in an Excel file. Another Excel file traces the examination process. It provides dates and times for all steps of the study procedure as well as personal codes of SAHR team members who completed procedures with the individual. It also includes dates of forwarding blood and urine samples to the laboratory and dates of getting their results. A special Excel macro transforms the individual files into two summary files named List#1 and List#2. The first file documents for the whole sample the process of recruitment that ends with successful recruitment, refusal (with or without stated reason), or inability to get in contact with the potential participant. The second file documents progress in interviews and medical testing.

Initially, the interview data are recorded on paper questionnaire forms. Afterwards, they are computerized two times and checked for inconsistencies between the two entries. The final interview data are stored in a SAS file. In this file, each individual's record contains 547 variables. Results of biomedical tests (except for Holter monitoring) are stored in the second SAS file after decoding of office ECG records and receiving laboratory results. In this file, each record contains 120 variables. Parameters of Holter series are computerized in the third file, which contains 71 variables. Personal IDNs allow merging of these data files. In addition, folders are set up with individuals' Holter binary files, RR-interval ASCII files, and JPG files with photographs.

The data files are further checked at SRCPM and at MPIDR. These checks include analysis for correct filtering through the questionnaire, checks of variable ranges, as well as some simple tabulations, correlations, and regressions.

### Follow-up and re-interview

The study participants are followed up by a phone call 14–18 months after the examination and by a trace of their vital status via residential registers. The follow-up phone call is to check whether they are still present at their registered places of residence and to update their vital and health status. Participants or their relatives are asked to provide brief information about any hospitalization or onset or aggravation of serious diseases experienced since the last contact. The most reliable information about participants' deaths and changes of location is obtained from residential registers. Such information usually has a lag time of 12 to 15 months.

For an average study participant, the re-interview will take place about 2.5 years after the first interview. Several questions address health events experienced since the baseline examination. A brief questionnaire includes seven modules replicating the baseline survey and one additional module highlighting attitudes towards aging (Table 4). The latter module consists of five questions adopted from the Philadelphia Geriatric Center Morale Scale [110,111]. It shows whether a person perceives his (her) own aging in an optimistic or pessimistic way.

**Table 4:** Modules of the brief questionnaire for the re-examination survey

Module 1. Information about the interview	Module 2. Marital status and economic conditions
Module 3. Health and quality of life	Module 4. Health behaviors
Module 5. Functional status	Module 6. Major life events
Module 7. Locus of control and depression	Module 8. Attitudes towards aging

If the study participant dies between the first and second interviews, the team will try to reach a proxy informant for preliminary information about the date and cause of death. The precise information about deaths will later be obtained from the residential registry.

The third column of Table 3 indicates several biomedical tests that will be administered for a second time during the re-examination: anthropometric measurements, grip strength, and office BP.

### Statistical analysis

The study data will provide a rich array of biomarkers collected from the general Moscow population. Despite the relatively small sample size, these data will open the door to numerous research opportunities.

Three principal types of statistical analysis will be carried out: descriptive and cross-sectional analyses, longitudinal analyses linking morbidity and mortality events and health changes with baseline predictors, and within-individual analyses of Holter time series.

As most of the collected biomarkers and some questionnaire indicators are newly obtained for the Russian population, descriptive and cross-sectional analyses of the baseline data will be important. They will highlight levels of these respective variables, show their variation across age, sex, and other dimensions and enable comparison with equivalent data from other populations. In particular, there is clear potential for comparison with the Cardiovascular Health Study and the MacArthur Study on Successful Aging in the United States, the Taiwan Social Environment and Biomarkers of Aging Study, the ELSA, the two Danish twin studies (LSADT and MADT), and the Eastern European HAPIEE study.

The analyses of associations will be focused on links within and between four major data blocks: measures of subjective and objective health, characteristics of socio-demographic status and wealth, characteristics of psychological stress, and biomarkers including Holter parameters. The block of health measures includes reported general health, reported diseases and invalidity; SF36 summary measures of physical functioning such as role limitations and bodily pain; objective cognitive and physical performance indicators such as MMSE, recall scores, and hand grip strength. The socio-demographic status is described by education, relative and absolute income, socio-occupational position, marital status, perceived relative wealth, proportion of income spent on food, and affordable expenses. Psychological status is characterized by the SF36 measures of role limitation due to emotional health, vitality, mental health, and social functioning; Cohen's stress scale; locus of control questions; battery on stress at work, at home, and financial stress; cynical distrust scale; depression scale; and coping with stress scale.

Finally, the block of biomarkers includes conventional cardiovascular risk factors (BP, lipid levels, and anthropometry); biochemical markers of allostatic load; conventional cardiac symptoms (Rose Angina, office heart rate, and ischemia signs on office ECG); ambulatory BP and especially its morning elevation; and major Holter markers (heart rate and its extremes; time and frequency domain parameters of heart rate variability; heart rate characteristics of sleep, circadian variation, and sleep-awake transition; tachycardia and bradicardia; prevalence of ECG distortions, and episodes of arrhythmia and ischemia.

The first cross-sectional analyses will assess links between subjective and objective health, between health and biomarkers; between health, socio-demographic characteristics and biomarkers; and between health, psychological stress and biomarkers. Among biomarkers, special attention will be paid to Holter-based variables. Analyses will be focused on their additional value and on locating the most informative and the best predicting parameters. The OLS, logit, ordinal logit, and multiple logit regressions as well as simultaneous equation models will make up the main statistical work to be performed.

The re-examination survey and the follow-up of study participants will provide data on mortality and morbidity events as well as on changes in health, disease, cognitive and physical performance variables registered by the re-examination survey. These data will be used for longitudinal analyses by means of proportional hazard and Poisson regressions. Preliminary estimates show that during the period between the baseline and the re-examination surveys about 150 deaths and about 100 serious health events can be expected. Information about the changes in health will be available for the whole sample. An additional follow-up of the SAHR participants for five years after the end of the Survey in 2011 would increase the number of observed death events to 560–580.

Use of aggregate scores instead of the original variables will be a reasonable option for many analyses. This approach enables one to reduce dimensions and to increase the statistical power of data without significant loss of explanatory capacity. SAHR data permit using many pre-defined questionnaire scores based either on batteries of questions (MMSE score, stress and depression scores) or on sets of biomarkers (the allostatic load score, Framingham score, and others). Additional scores can be constructed from the original variables on the basis of regression and factor analyses.

A special section of analyses will be devoted to the continuous ECG time series. First, mathematical procedures and algorithms for robust estimation of RR series from the ECG signal and computation of frequency domain indices will be worked out. At a later stage, statistical models for automated estimates and informative measurements from the 24-hour series will be explored. In particular, automated recognition of awake-sleep and the sleep-awake transitions is one of the priority problems. This can be done by exploiting the statistical techniques provided by hidden Markov models. The ability of such models to effectively segment ECG trajectories has been proven in several studies [112,113].

## Discussion

SAHR will produce unique insights into the determinants of chronic conditions and ill health in Russia, a region where mortality has long been very high and has been further increasing over the last two decades. Reasons for this unusual trend are poorly understood, especially with regard to older ages where chronic health conditions and biological factors are of greater importance. So far, even some very basic objective data on the physical and cognitive health of Russians are not available. The SAHR aims at closing this gap by providing a large amount of objective measurements and biomedical data. The study's collection of biomarkers combines well-established biomarkers with newer markers of allostatic load that presumably measure cumulative stress and physiological toll. The novel dynamic data of 24-hour Holter monitoring allows to evaluate the functioning of the cardiac system, as well as to see how the system responds to routines and challenges of everyday life.

As in many other studies rich in medical data, the SAHR has a relatively small sample size. Although it represents a disadvantage (especially for the longitudinal analyses of mortality and morbidity), it can be (at least partly) treated by the use of changes in health as the dependent variable and/or by the use of aggregate biomarker or questionnaire scores as the independent variables.

The following three data problems do not influence any analysis within the study, but could cause difficulties for comparisons with external data. First, SAHR participants represent the Moscow population, yet that population differs in some respects from the entire Russian population. Most importantly, Moscow has a greater proportion of people with higher education and those with higher income. Use of weights can (at least partly) treat this problem and bring the SAHR sample closer to the national population.

Second, the SAHR faces a well-known problem related to a certain selectivity of participants to the sample. For example, response rates tend to be higher in women and in people with higher education. We partly compensate for this problem by a moderate over-sampling of the under-represented groups and by use of appropriate weights for international comparisons.

Third, any *quantitative *comparison of Holter parameters with equivalent values from other studies should be made with caution and accompanied by checks of comparability. The latter depend on Holter systems, algorithms for detection of R peaks, estimation of HRV parameters and ECG patterns. Some Holter parameters (for example, overall heart rate) are quite robust, but many parameters are sensitive to these factors. Ideally, Holter biomarkers should be computed by one and the same software from ECG files of different origin.

The SAHR will provide the most comprehensive set of new biomedical and questionnaire data for the Russian population. These data will enhance research for disclosing mechanisms underlying the heavy burden of death and disease among Muscovites aged 55 and older. The study will also contribute to the understanding of general relationships between objective life conditions, psychological stress, and biological and health changes in humans. Analysis of the Holter series will provide new opportunities to observe continuous functioning of the heart and its regulation by the autonomous nervous system under conditions of everyday life. These data have potential to add further value to the known biomarkers and will help researchers take the next step towards health monitoring systems of the future.

## Competing interests

The authors declare that they have no competing interests.

## Authors' contributions

MS participated in the design of the study, coordinated the study at MPIDR, provided expertise on ECG and Holter, and other biomedical issues, outlined the paper, and helped in its writing. SS participated in the design of the study, coordinated the study work at SRCPM, and helped to draft the paper. VMS participated in the design of the study, developed a core part of its questionnaire, carried out preliminary statistical analyses, and prepared the final paper. VM coordinated laboratory works for the study and drafted the paper. AD coordinated selection and recruitment of participants to the study, carried out preliminary statistical analyses, and helped in drafting the paper. EA coordinated data processing and helped to draft the manuscript. DJ carried out preliminary analyses of Holter series and helped in drafting the paper. JWV coordinated the whole project development and implementation and helped in finalizing the paper. All authors have read and approved the final manuscript.

## List of abbreviations

ACC: American College of Cardiology; AHA: American Heart Association; BP: Blood pressure; C: Cholesterol; CRP: C-reactive protein; DBP: Diastolic blood pressure; DHEA-S: Dehydroepiandosterone sulfate; ECG: Electrocardiogram; ECLT: Euglobulin fraction Clot Lysis Time; ELSA: English Longitudinal Study of Aging; GGT: Gamma-glutamyl transferase; HAPIEE: Health, Alcohol and Psychological Factors In Eastern Europe; Hb: Haemoglobin; HbA1c: Glycosylated haemoglobin; HDL: High-density lipoprotein; HF: Power component in the high frequency range 0.15–0.4 Hz; HR: Heart rate; HRV: Heart rate variability; IL-6: Interleukin-6; LDL: Low-density lipoprotein; LF: Power component in the low frequency range 0.04–0.15 Hz; LSADT: Longitudinal Study of Aging Danish Twins; LRC: Lipid Research Clinic; MADT: Study of Middle-Aged Danish Twins; MPIDR: Max Planck Institute for Demographic Research; MONICA: Multinational MONItoring of trends and determinants in CArdiovascular disease; MPS: Moscow Pilot Study; OLS: Ordinary least squares; pNN50: Percentage of adjacent normal-to-normal that vary by more than 50 ms; POWER: Spectral power for all normal-to-normal intervals in the frequency range ≤ 0.4 Hz; RLMS: The Russian Longitudinal Monitoring Study; rMSSD: The square root of the mean of the sum of successive differences of normal-to-normal intervals; SAS: Statistical Analysis System; SAHR: Survey on Stress Aging and Health in Russia; SDANN: Standard deviation of the averages of normal-to-normal intervals within all 5-minute segments; SDNN: Standard deviation of all normal-to-normal intervals; SF-36: 36-item Short Form Health Survey; SBP: Systolic blood pressure; SRCPM: State Research Centre for Preventive Medicine; VLF: Power component in the very low frequency range ≤ 0.04 Hz; WBC: White blood cells.

## Pre-publication history

The pre-publication history for this paper can be accessed here:


